# Biomarkers in critical care nutrition

**DOI:** 10.1186/s13054-020-03208-7

**Published:** 2020-08-12

**Authors:** Christian Stoppe, Sebastian Wendt, Nilesh M. Mehta, Charlene Compher, Jean-Charles Preiser, Daren K. Heyland, Arnold S. Kristof

**Affiliations:** 1 3CARE—Cardiovascular Critical Care & Anesthesia Evaluation and Research, Aachen, Germany; 2 Department of Anesthesiology, University Hospital RWTH Aachen, Pauwelsstrasse 30, 52074 Aachen, Germany; 3 Department of Anesthesiology, Critical Care and Pain Medicine, Division of Critical Care Medicine, Boston Children’s Hospital, Harvard Medical School, Boston, MA USA; 4 Department of Biobehavioral Health Science, University of Pennsylvania and Clinical Nutrition Support Service, Hospital of the University of Pennsylvania, Philadelphia, PA USA; 5 Erasme University Hospital, Université Libre de Bruxelles, 808 route de Lennik, B-1070 Brussels, Belgium; 6 Department of Critical Care Medicine, Queen’s University, Angada 4, Kingston, ON K7L 2V7 Canada; 7 Clinical Evaluation Research Unit, Kingston General Hospital, Angada 4, Kingston, ON K7L 2V7 Canada; 8 Meakins-Christie Laboratories and Translational Research in Respiratory Diseases Program, Faculty of Medicine, Departments of Medicine and Critical Care, Research Institute of the McGill University Health Centre, 1001 Décarie Blvd., EM3.2219, Montreal, QC H4A 3J1 Canada

**Keywords:** Nutrition, Biomarker, Metabolism, Critical care

## Abstract

The goal of nutrition support is to provide the substrates required to match the bioenergetic needs of the patient and promote the net synthesis of macromolecules required for the preservation of lean mass, organ function, and immunity. Contemporary observational studies have exposed the pervasive undernutrition of critically ill patients and its association with adverse clinical outcomes. The intuitive hypothesis is that optimization of nutrition delivery should improve ICU clinical outcomes. It is therefore surprising that multiple large randomized controlled trials have failed to demonstrate the clinical benefit of restoring or maximizing nutrient intake. This may be in part due to the absence of biological markers that identify patients who are most likely to benefit from nutrition interventions and that monitor the effects of nutrition support. Here, we discuss the need for practical risk stratification tools in critical care nutrition, a proposed rationale for targeted biomarker development, and potential approaches that can be adopted for biomarker identification and validation in the field.

## Background

The delivery of nutritional substrates and their use for anabolic metabolism are thought to play key salutary roles in the natural history of critical illness. Observational studies in children [1, 2] and adults [3–8] have consistently shown that energy and protein deficit is strongly associated with increased complications and mortality and that factors associated with metabolic vulnerability (e.g., rapid weight loss) might identify those patients most likely to benefit from optimal nutrition support [9, 10]. Experts have therefore recommended enteral or parenteral formulations that supplement macronutrients, especially protein, at levels thought to limit catabolism [11–15]. Despite strong observational data, however, the field has struggled with the failure of randomized controlled trials (RCTs) to demonstrate a clear benefit from nutrition support [16].

To explain this discrepancy, many have cited potential weaknesses in study designs [17–19] that have not accounted for heterogeneity in treatment effect (HTE) or biases that are difficult or impossible to discern [20]. Heterogeneity might arise from differences in disease severity and prognosis among individuals [21]. That is, the effect of an intervention may not be detected in an RCT if many of the subjects are at low risk of developing the endpoint of interest. This is especially problematic in ICU nutrition RCTs which likely included many patients with low nutritional risk [16]. Heterogeneity might also arise from individual differences in responses to nutrition support interventions. Mechanisms might include maladaptive metabolic changes (e.g., anabolic resistance) [22] that prevent a salutary response, or the reversal of otherwise adaptive responses to nutrient restriction that might have been beneficial (e.g., autophagy) [23, 24]. Finally, the complex interplay between macronutrient dose, timing, and route of administration is poorly understood and necessitates a more nuanced precision approach to the design of RCTs in critical care nutrition. Here, we provide a rationale for the use of biomarkers to address these gaps, a summary of the current state of the art, and a proposed approach for their development and implementation in critical care nutrition.

## The need for biomarkers in critical care nutrition

Biomarkers (see Table 1) can be applied as diagnostic or prognostic tools for clinical decision-making or for risk stratification in clinical trials [25, 26]. Ideally, they closely reflect the biological mechanisms involved in disease pathogenesis and response to therapy [26]. Prognostic markers are especially useful in clinical trials for the identification of patients at high risk of developing the outcome of interest. For instance, an ongoing trial of high- vs. low-dose protein in the critically ill (EFFORT; NCT03160547) was enriched for patients at high nutritional risk (i.e., low or high BMI, frailty, diagnosed malnutrition, mechanical ventilation) [27]. Predictive biomarkers reflect heterogeneity in individual responses to an intervention. For instance, in the critically ill, protein biomarker panels were identified that classify patients with the adult respiratory distress syndrome (ARDS) who are most likely to benefit from positive end-expiratory pressure (PEEP) or conservative fluid strategies [28, 29]. Biomarkers might similarly predict the magnitude, kinetics, and heterogeneity of metabolic responses to nutrition support, thereby addressing crucial questions regarding dose, timing, and nutrient substrates used during nutrition support.

**Table 1:** Definitions

Biomarker	Measurable indicator of biological processes, pathogenic states, or pharmacologic responses to therapeutic interventions
Prognostic biomarker	Reflects an individual’s risk of developing an outcome or endpoint of interest
Predictive biomarker	Reflects the likelihood that an individual can respond to an intervention of interest
Surrogate biomarker	Biomarker that correlates with clinical endpoints reflecting patient well-being or survival
Enrichment	The use of prognostic or predictive tools to choose or analyze a study sample that maximizes the likelihood of establishing a treatment effect
Endotypes	Subsets of patients classified by common pathobiological mechanisms

In the ICU, diverse biological processes might explain why patients respond differently to nutrition interventions (i.e., metabolic heterogeneity), and many have been outlined by a recent expert panel calling for new patient-centered approaches to metabolic support in the critically ill [30]. For instance, phases of metabolic response have been well documented during critical illness, and, for individual patients, these can differ in extent and timing [22]. Nutrient availability or utilization can be altered by gut bacteria, enteric absorption, utilization by tissues, and alterations in cellular adaptive processes. Additional sources of variability include metabolic, genetic, or epigenetic drivers of disease susceptibility and progression, as well as underlying etiology of illness and severity of organ dysfunction [31]. Biomarkers that can be used to select patients at high risk of adverse clinical outcome if nutritional needs are not met (i.e., prognostic), or those that predict beneficial responses to nutrition support (i.e., predictive), would greatly facilitate the design of trials by enriching for those patients most likely to benefit.

## Biomarkers in critical care nutrition: current state of the art

The need to better define nutritional risk and metabolic heterogeneity, as well as to assess responses to nutrition interventions, led to the development of several tools in the field. Since organ dysfunction is the primary predictor of clinical outcomes in the critically ill, we focus here on biomarkers that are most likely to reflect metabolic responses to nutrition at the organ or whole-body level (Table 2).

**Table 2:** Unidimensional biomarkers in nutrition

Biomarker	Indicator of nutritional risk	Valid measure of metabolic response to nutrition	Indicates a biological mechanism related to the intervention and clinical outcomes	Feasible for use in ICU clinical practice or trials	Additional procedures and samples required
Baseline clinical parameters: BMI, NUTRIC Score	Yes	No	No	Yes	None
Whole-body protein balance	Unknown	Yes	Yes	No	Isotope infusion; frequent sampling of blood and exhaled breath
Nitrogen balance	Unknown	No	Yes	Yes	Collection of urine over 6–24 h and blood sampling
Insulin resistance	Unknown	Unknown	Yes	Yes	None
Albumin, pre-albumin	Yes	No	Unknown	Yes	Blood sampling
Body composition (skeletal muscle ultrasound, CT)	Yes	Unknown	Yes	Unknown	Imaging
Markers of inflammation (IL-6, CRP)	Yes (IL-6)	Unknown	Yes	Yes	Plasma ELISA, PaxGene PCR

### Tools for risk stratification at baseline

The Nutrition Risk in the Critically Ill score (NUTRIC) is a prognostic tool developed by Heyland et al. [10, 32]. The parameters derived, including the inflammatory cytokine interleukin-6 (IL-6), are related to co-morbidity and severity of illness, and thus may be used to enrich study samples for those patients who are more likely to survive if their estimated caloric or protein needs are met. A post hoc analysis of the TOP-UP trial suggested that this may apply in prospective fashion [33], but similar discriminative performance was not replicated in another recent RCT [34], and additional validation is pending [27].

### Biomarkers of whole-body metabolism

Of the measures that reflect whole-body nutrient absorption and metabolic responses, the most robust are measures of protein synthesis and breakdown [35]. In non-ICU settings, “whole-body protein balance,” as assessed by stable isotope tracer infusion, was a physiological indicator of metabolic response to nutrition or other anabolic interventions (e.g., insulin therapy) [36]. In the critically ill, protein balance was increased by energy intake, and the initiation of enteral feeding promoted anabolism [37, 38]. Although this method is robust, its complexity renders it costly and impractical as a clinical biomarker in large RCTs. Similar limitations appear in the pediatric population [39].

In contrast to whole-body protein balance, the calculation of nitrogen balance *estimates* the net difference between protein synthesis and breakdown [40, 41]. Nitrogen balance has been used to establish dietary protein requirements in healthy subjects [42], but performs poorly in critically ill adults [43, 44] and children [45, 46]. Sources of variability include rapid changes in total body water, as well as urinary and extra-urinary losses of nitrogen. Nonetheless, higher protein intake appeared to increase [47, 48], or was associated with higher [49], nitrogen balance in the critically ill but did not appear to predict clinical outcomes. Emerging markers of whole-body metabolism include measures of body composition [50] and are discussed further in the next section.

Although changes in whole-body protein, nitrogen balance, or body composition might be useful to monitor metabolic responses in the general population [35, 50], little is known regarding their relationship to clinical outcomes in the ICU. Other putative markers of nutritional status, substrate availability, and metabolism include albumin, pre-albumin, retinal binding protein, transthyretin, and transferrin [51–54]. Their levels are not, however, acutely altered by nutrition interventions, nor do they reliably predict clinical outcomes.

### Systemic markers of inflammation

One hypothesis underpinning the rationale for nutrition support is that the reversal of catabolism might attenuate inflammation or promote tissue regeneration, which are both linked to organ failure and mortality. In fact, the best predictors of mortality in the critically ill (e.g., APACHE, SOFA) include clinical measures of systemic inflammation and organ injury [55], suggesting that attenuation of the latter might improve clinical outcomes. Similarly, the reversal of skeletal muscle catabolism might prevent muscle atrophy during critical illness and improve functional outcomes [56–58]. A recent review of ICU nutrition RCTs identified a need for studies with pre-planned evaluations of inflammatory mediators as potential biomarkers of nutrition interventions [16].

Little is known regarding how exogenous nutrition affects tissue injury or repair during critical illness, but biomarkers of systemic inflammation and organ injury have been well characterized [59]. These include markers of oxidative and nitrosative stress, complement activation, inflammation (i.e., pro-inflammatory cytokines), and organ dysfunction (e.g., anaerobic metabolism, cell death) [60]. In addition, patients with severe systemic inflammation can exhibit a compensatory anti-inflammatory response syndrome (CARS), which is associated with immune paralysis and reduced survival [61]. Single-parameter (e.g., BAL neutrophils for VAP [62]) or multiplexed (e.g., cytokine panel for ARDS [29, 62]) measures of inflammation can be used as prognostic biomarkers (reviewed in [63]), but their ability to predict responses to nutrition or metabolic heterogeneity in ICU patients has not yet been explored. Emerging studies in non-ICU populations suggest that systemic markers are promising candidates. In patients undergoing cardiac surgery, amino acid supplementation attenuated bypass-related induction of interleukin-6 (IL-6) and tumor necrosis factor-α (TNF-α) [64]. Elevated plasma IL-6 levels were associated with benefit from nutrition interventions in the ICU setting [10], but its prospective use as a monitoring or stratification tool has not been fully explored. These emerging studies suggest that the serial measure of inflammatory markers over time may be useful in determining the timing of nutrition interventions and in identifying patients who will benefit.

In summary, current biomarkers (Table 2) are either difficult to widely implement or fail to fully reflect the biological mechanisms that predict susceptibility or physiological responses to nutrition interventions [65]. To date, they have not been used as prognostic or predictive tools in contemporary clinical trials. In addition, unidimensional markers (e.g., IL-6, pro-calcitonin) have failed to confer prognostic classification in RCTs evaluating other ICU-related pathologies, such as sepsis or ARDS [63]. There is a pressing need for alternative approaches that can be exploited to better understand and monitor the biological mechanisms related to nutritional risk and metabolic heterogeneity.

## Developing effective biomarkers of nutrition

Unlike the hypothesis-based (i.e., biased, targeted) use of unidimensional biomarkers, untargeted multidimensional approaches better encompass the spectrum of known and unknown biological mechanisms that ultimately define endotypes. Endotypes are subsets of patients classified by common pathobiological mechanisms [26], and can be used to classify patients according to their risk of developing an outcome of interest (prognostic enrichment), or responsiveness to an intervention (predictive enrichment) [21, 66]. These untargeted approaches can be guided by our evolving understanding of the basic mechanisms that mediate cellular nutrient sensing and that drive metabolism, inflammation, and repair in the critically ill.

### Biological responses to nutrient restriction (starvation) and supplementation (feeding)

Critically ill patients with organ dysfunction are catabolic on admission to the ICU [37]. This results from pathophysiological drivers (e.g., systemic inflammation, stress hormones) as well as nutrient restriction both preceding and after ICU admission [5, 6]. Like the described metabolic adaptations to critical illness [22], cellular adaptations to nutrient restriction can occur rapidly and are mediated by proteins that sense the levels of anabolic substrates or cellular energy. For example, the protein kinase “mechanistic target of rapamycin” (mTOR) coordinates a signaling hub linking nutrient or energy availability (e.g., amino acids) with cellular adaptations to nutrient restriction (e.g., autophagy), gene expression, and epigenetic mechanisms that predict prolonged changes in cellular function [67]. In fact, transporters or intracellular receptors can bind or transport single essential amino acids (e.g., leucine) which control mTOR anabolic activity in pharmacological fashion [68, 69]. Heterogeneity in these responses might arise from genetic variability or from the prolonged epigenetic shifts that can cause sustained dysregulation of bioenergetics and metabolism [67]. Other related cellular nutrient and energy sensors include “general control non-derepressable-2” (GCN2) [70] and the liver kinase B1/5′ AMP-activated protein kinase (LKB1/AMPK) pathway, which sense metabolic stress and control adaptive bioenergetic responses.

Population heterogeneity in the extent and kinetics of nutrient-related metabolic responses can only be defined by detailed multidimensional assays and their repeated measures over time. Several suppositions can nonetheless be made that pertain to biomarker development. First, tissues differ in their responses to metabolic stress, and their responses to nutrient availability are likely to vary. For instance, the profound protein catabolism and atrophy needed for mobilization of amino acids from skeletal muscle could be an adaptive mechanism to support other essential functions (e.g., immunity) required during severe illness [71]. Second, the dysregulated cellular metabolism observed during critical illness may subvert the use of available nutrients for ATP production or protein synthesis, and untimely nutrient delivery may inhibit adaptive responses [22]. Third, genetic and epigenetic variation in mechanisms that mediate energy and substrate availability, sensing, and metabolic responses may account for inter-subject variability. Finally, nutrient restriction or systemic inflammation may induce epigenetic responses leading to sustained alterations in the metabolism that cannot be immediately overcome by nutrition support. Detailed profiling of these changes in patients over time would shed important insights into the range and heterogeneity of responses to nutrient availability, as well as the optimal timing for initiating or withholding nutrition support. Time-sensitive factors include anabolic resistance, microbiome diversity, organ dysfunction, and endocrine stress responses [22].

Several features of intracellular metabolic sensing pathways (e.g., mTOR) render them potentially useful as biomarkers. They can exhibit pharmacological ligand/receptor properties and dose-response characteristics. In some cases, tissue availability of nutrition-derived substrates (e.g., leucine) correlated with dietary intake [72, 73]. Nutrient sensing mechanisms also mediate important organ-specific functions, including skeletal muscle catabolism [74] and immune dysfunction [75–77], which can be measured and employed as surrogate outcomes. Finally, intracellular sensors of metabolism control the metabolic shifts in response to systemic inflammation or nutrient availability that drive the innate or adaptive immune repertoire [67, 78, 79]. However, the evaluation of enzymatic activity, post-translational modification, or levels of intracellular proteins is impractical in the clinical setting and exhibits suboptimal dynamic range and reproducibility. Alternatively, multiplexed assays for the biological effector mechanisms initiated by nutrient sensing (e.g., patterns of gene expression) can be performed on samples that are easy to obtain at low risk to the patient and that can be potentially adopted as point-of-care clinical tests.

### Rationale for multidimensional approaches to biomarker development in critical care nutrition

**Fig. 1:**
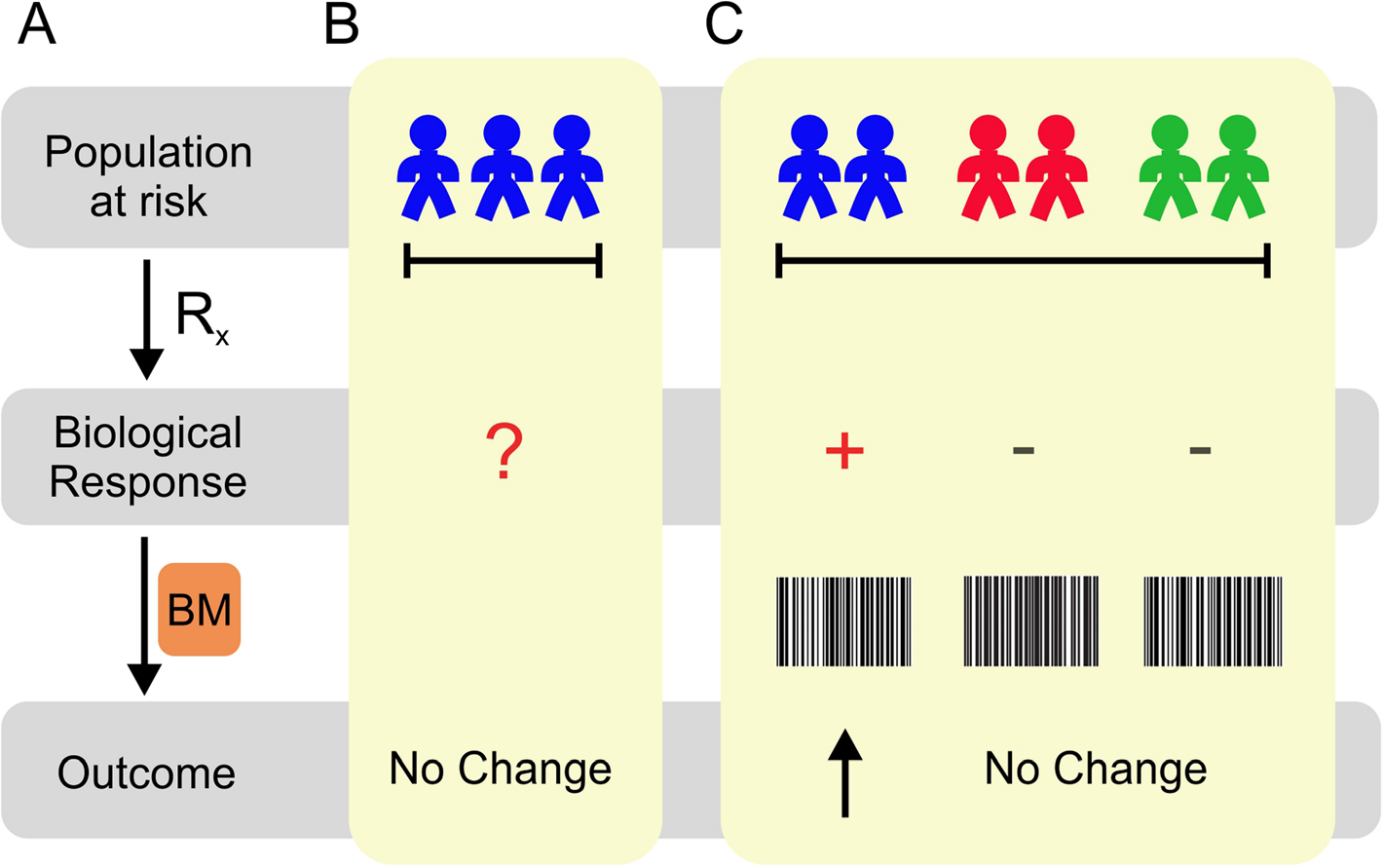
**a** Clinical decision-making and RCTs operate on the hypothesis that an individual or population at risk is administered a therapeutic intervention that leads to a salutary biological response and a better health outcome (gray). Biomarkers (BM; orange) are used to monitor therapeutic responses or to define subsets of the population most likely to benefit from the intervention (Rx). **b** RCTs in the critical care nutrition field have assumed homogeneous (blue) nutritional risk, and they have not exploited biomarkers (red interrogation mark) to target patients at risk or to time nutritional interventions. Outcomes have been equivocal or difficult to interpret. **c** ICU patients exhibit metabolic heterogeneity (mixed colors), and multidimensional assays (bar codes) are best to capture the patients’ endotypes (e.g., blue) that predict clinical responses to nutrition support (red plus sign). Multidimensional biomarkers can be used to limit enrollment to those patients most likely to benefit (blue) or to enrich the results by classifying patients during post hoc analyses. Biomarker panels can be generated using new omics technologies that measure biological properties that are highly linked to nutrition and metabolism. Primary candidates are genomic, transcriptomic, epigenomic, and microbiome-based assays, which can then be reduced and implemented in non-invasive point-of-care assays

Unlike single analytes, multidimensional markers are more likely to delineate biological signaling networks related to intervention and outcome of interest, or to define endotypes of nutritional responsiveness (Fig. 1) [80–83]. The predictive ability of a surrogate biomarker is driven by the proportion of the treatment effect (PTE) that is mediated by the measured analyte(s) (reviewed in [26]). A biomarker would thus exhibit a high PTE if it was a biological mediator of the response to the intervention and of the clinical outcome of interest. For instance, as a surrogate biomarker, the detection of *Streptococcus pneumoniae* in the blood and its subsequent eradication would exhibit a high PTE when testing the effect of an appropriate antibiotic on mortality. Unidimensional markers have not captured the biological complexity of critical illness, nor have they effectively classified prognostic groups. Examples include the co-syntropin stimulation test and IL-6, respectively, which were employed unsuccessfully as potential prognostic classifiers in recent RCTs (reviewed in [63]). In contrast, multiplexed assays, or biomarker panels, resemble barcodes that can more effectively classify patients into subgroups according to biological factors that predict therapeutic or prognostic effects (Fig. 1c). Some examples include prognostic panels developed for pediatric and adult sepsis [84, 85]. It remains less clear, however, how well these tools monitor therapeutic interventions.

The development of multidimensional biomarkers is now feasible, in part due to advances in sequencing technologies, detailed annotation of DNA or RNA sequences, and evolving bioinformatic tools including machine learning approaches. Metadata using whole “omics” analyses in the clinical setting can be reduced to construct biomarker panels that exhibit high PTE and that capture patient heterogeneity [85, 86]. Untargeted approaches also provide value by revealing previously unknown (i.e., emergent) mechanisms of disease that can inform the development of novel therapeutics. For example, combined genetic, transcriptomic, and epigenomic analyses have revealed metabolic pathways that drive inflammation in response to microbial products [87], as well as metabolically driven epigenetic mechanisms by which inflammation can be altered in sustained fashion (i.e., trained immunity) [88]. These prolonged epigenetic modifications likely contribute to immune or metabolic dysfunction and might thereby modify the effects of dose and timing of nutrition in the critically ill.

### Assays for biomarker identification in critical care nutrition

We propose that the most promising assays are those related to nutrient availability and metabolism in easily accessible tissues or fluids and that exploit technologies which can be rapidly adapted to the clinical setting (Table 3).

**Table 3:** Promising mechanisms for multidimensional biomarker development

Mechanism	Potentially influenced by nutrient signaling pathways	Might reflect susceptibility to nutrition intervention	Could reflect response to nutrition intervention	Assays	Currently feasible for point of care
Genetic	No	Yes	No	Blood or saliva NGS, PCR	No
Transcriptomic	Yes	Yes	Yes	Blood RNA-seq, PCR	Yes
Epigenomic	Yes	Yes	Yes	Blood ATAC-seq, bisulfite-seq	No
Proteomic	Yes	Yes	Yes	Blood mass spec, ELISA, Western blot	Yes
Metabolomic	Yes	Yes	Yes	Blood or breath volatiles mass spec, HPLC	No
Microbiome	Yes	Yes	Yes	Stool, respiratory secretions; 16S sequencing, metagenomics	No

#### DNA sequencing

Contemporary technologies can detect the sequences of all genes in an individual subject and then derive endotypes related to the likelihood of metabolic response to nutrition support. DNA is easily obtained from clinical samples and can be interrogated by point-of-care amplification assays. Although genomic variants may contribute to understanding susceptibility or prognosis, they are static and cannot reflect tissue-specific changes or therapeutic responses. Moreover, genetic variants tend to account for a low proportion of the treatment effect (PTE), since environmental factors (e.g., infection, medications) heavily influence interventions and outcomes in critical illness. Nonetheless, biomarker candidates have emerged from genomic analyses in other domains of critical illness. For instance, genetic variants correlated with greater mortality risk reduction attributed to activated protein C [89].

#### Assessment of mRNA levels and epigenetic modifications

In contrast to genomic DNA, RNA expression and epigenomic modifications are dynamic and can be directly altered by changes in metabolism or nutrient signaling mechanisms [90, 91]. In addition, the cellular metabolic shifts that occur during critical illness can alter the levels of substrates (e.g., α-ketoglutarate, *S*-adenosyl methionine) and enzymes (e.g., DNA methylases, histone acetyl transferases) used for chromatin modification and selective gene induction or silencing. Whereas RNA-seq is the current standard for characterizing the whole transcriptome, technologies for genome-wide epigenomic assessment are only now emerging [92, 93]. Once reduced to biomarker panels, analytes derived from transcriptomic and epigenomic metadata can be tested in multiplex PCR-based panels [85]. The measurement of whole blood or peripheral blood mononuclear cell mRNA levels can be implemented in the clinical setting and in the context of RCTs [63]. Technologies for the standardized measurement of DNA or RNA are available in clinical laboratories or as point-of-care tests. In contrast, most epigenomic assays either require live cells (e.g., ATAC-seq) or large sample volumes (e.g., bisulfite sequencing), indicating, for the time being, that their use might be restricted to the enrichment of RNA-based panels during the biomarker identification phase.

#### Proteomic and metabolomic assays

Whereas proteomic and metabolomic assays are promising tools for biomarker development, their annotation and interpretation are less well-developed than those for evaluation of DNA and RNA. Nonetheless, new technologies that permit the evaluation of large numbers of proteins in the same sample have been used to establish endotypes in critical illness [28]. Metabolomic approaches to identify prognostic indicators were employed in retrospective critical care cohorts, but their ability to prospectively predict response to nutritional interventions has not been systematically evaluated (reviewed in [94]). Both proteins and metabolites can potentially be measured in point-of-care mass spectrometry-based devices.

#### Microbiome

Gut microbiome diversity, as assessed by 16S microbial sequencing or metagenomics, is another candidate multidimensional biomarker that is significantly altered during critical illness [95–97], can be modified by nutrient intake [98], and leads to adaptive or pathophysiological mechanisms that may alter clinical outcomes [31]. In agreement, gut and airway microbial diversities were inversely correlated with the severity of illness and mortality [97]. More studies are needed, however, to explore how microbial diversity is affected by timing, dose, and route of nutrition support, and how these changes impact upon patient outcomes [98].

#### Imaging

There has been an emerging interest in the evaluation of the skeletal muscle or lean body mass as an indicator of response to nutrition support and functional outcomes in ICU survivors [50, 99]. These include imaging modalities that can be performed at the bedside using portable technologies and include ultrasound evaluation of skeletal muscle thickness [100] or fat-free mass [101] and CT evaluation of paravertebral or limb fat to muscle ratio [56]. For instance, a well-designed ultrasound protocol allows clinicians to assess quantitative and qualitative changes in the skeletal muscle in mechanically ventilated patients [102]. The results of ongoing clinical trials evaluating the effect of protein nutrition and/or exercise on functional recovery in the critically ill are pending (e.g., EFFORT sub-study, NEXIS [27, 103]). In addition to bedside approaches, nuclear imaging techniques that measure tissue metabolite absorption and kinetics (e.g., 2DG-PET for cancer) might be adapted to detect metabolic substrate uptake (e.g., leucine, phenylalanine) during nutrient restriction or supplementation at the organ or whole-body level. However, measures that require patient transport for lengthy imaging procedures are less viable options in the critically ill.

### Incorporating biomarker development into critical care nutrition research

Investigators and policymakers have outlined requirements for the development and adoption of biomarkers in clinical trial design [25]. For nutrition support, challenges include the complexity of formulations administered and the need to better define the biological mechanisms by which nutrient availability regulates cellular and organ function. Despite these caveats, the pharmacodynamic properties of complex nutritional interventions can be measured (e.g., whole-body metabolism, nutrient-sensing pathways) and can be exploited for biomarker development. Moreover, individual or limited mixtures of macro- and micro-nutrients are increasingly being tested in clinical trials for their immune-modifying effects (e.g., vitamin C, omega-3) [16, 104].

Given the current limitations in the field of critical care nutrition, collaborations between basic, translational, and clinical scientists in well-funded consortia are crucial to better understand the biological mechanisms related to nutrient metabolism in pre-clinical and clinical models. Prospective longitudinal cohorts that interrogate clinical data and banked samples for genetic and molecular analyses can be useful in identifying biomarker candidates. In the absence of existing observational cohorts, phase I and II trials provide an environment in which to prospectively measure the relationship between nutrition dose and the levels of putative biomarkers, to establish pharmacodynamic properties of nutrition support interventions, or to better define metabolic heterogeneity [105]. Effective approaches for the development of biomarkers and their incorporation within phase II and III clinical trials have been reviewed [105, 106]. The primary outcomes in phase II studies are usually measurable disease-related or physiological responses to an intervention (e.g., protein balance) and can be evaluated in small samples; their simultaneous correlation with multidimensional molecular analytes is a promising way to identify multiplex biomarkers of nutritional response. In addition, phase II trials are amenable to adaptive design, in which interim analyses of biomarker levels can be used to change randomization ratios, and thereby enrich for those patients most likely to respond to the intervention. Ultimately, biomarkers would contribute to the creation of core outcome sets that can be used to synthesize research across critical care nutrition trials [107].

## Conclusion

Nutrition research has been burdened by methodological and practical challenges that limit our ability to extract meaningful conclusions from clinical trials (reviewed in [108]). Current knowledge gaps and potential solutions are summarized in Table 4. Experts and regulators now propose the adoption of biomarker-based enrichment tools early in the process of trial design [25], as well as their incorporation in core outcome sets [86, 107]. There is therefore a pressing need for collaborative and standardized approaches to biomarker identification, validation, and implementation in nutrition RCTs. Multidisciplinary consortia would promote the rapid translation of new biomarker technologies, a departure from the current “one-size-fits-all” approach to trial design, and a new era of precision medicine in critical care nutrition.

**Table 4:** Current challenges and potential approaches to biomarker development in critical care nutrition

Challenges	Solutions
Understand biological mechanisms	Multidisciplinary consortia with basic, translational, and clinical scientists focused on pre-clinical and clinical mechanistic models of nutrition and metabolism.
Characterize metabolic heterogeneity and response to nutrition support with respect to: • Kinetics • Nutrition support modality • Dose	Longitudinal cohorts and/or phase I or II clinical trials with the collection of granular physiological and omics data that can be correlated with meaningful clinical endpoints. Emphasis is placed on genomic, transcriptomic, epigenomic, and microbiome patterns of metabolic response to nutrition support.
Identify diagnostic and prognostic biomarkers that can classify responses to nutrition support by: • Likelihood of response • Appropriate timing of initiation • Adequacy of modality and dose	The statistical reduction of multidimensional data sets collected over time into biomarker panels that can capture endotype-driven treatment effects and population heterogeneity, thereby permitting the design of “smart” trials.
Validate and implement biomarkers	Leverage detailed observational cohorts or multi-center RCT’s establish external validity, utility, and feasibility in large multi-center RCTs.
Foster the development of biomarker in critical care nutrition research and program development	Promote biomarker development in the early stages of pre-clinical and clinical study design and the adoption of biomarkers for risk stratification tools in clinical trials.
Overcome technological and economic barriers: • Assay complexity • Sampling • Cost	Focus on samples that can be easily obtained at low cost (e.g., blood) and assays that can be feasibly adopted as point-of-care tests (e.g., PCR, ELISA).

## Data Availability

Not applicable.
